# LanDis: the disease landscape explorer

**DOI:** 10.1038/s41431-023-01511-9

**Published:** 2024-01-10

**Authors:** Horacio Caniza, Juan J. Cáceres, Mateo Torres, Alberto Paccanaro

**Affiliations:** 1 Universidad Paraguayo Alemana de Ciencias Aplicadas, Facultad de Ciencias de la Ingeniería, San Lorenzo, Paraguay; 2 Department of Computer Science, Centre for Systems and Synthetic Biology, Royal Holloway University of London, Egham, UK; 3 Escola de Matemática Aplicada, Fundação Getúlio Vargas, Rio de Janeiro, Brazil

**Keywords:** Genome informatics, Genetics research, Data processing

## Abstract

From a network medicine perspective, a disease is the consequence of perturbations on the interactome. These perturbations tend to appear in a specific neighbourhood on the interactome, the disease module, and modules related to phenotypically similar diseases tend to be located in close-by regions. We present LanDis, a freely available web-based interactive tool (https://paccanarolab.org/landis) that allows domain experts, medical doctors and the larger scientific community to graphically navigate the interactome distances between the modules of over 44 million pairs of heritable diseases. The map-like interface provides detailed comparisons between pairs of diseases together with supporting evidence. Every disease in LanDis is linked to relevant entries in OMIM and UniProt, providing a starting point for in-depth analysis and an opportunity for novel insight into the aetiology of diseases as well as differential diagnosis.

In recent decades, our understanding of diseases and their causes has shifted from simple relationships between genes and diseases to more comprehensive models, which take into account the interplay of gene products through their multiple molecular interactions. The set of interactions between proteins can be summarised in a network, often referred to as interactome, where nodes represent proteins and links represent interactions between them. Studying diseases in the context of the human interactome has revealed that a disease’s causal genes tend to cluster in close-by regions—the disease module—and that diseases that share causal genes tend to exhibit phenotypical similarity [1]. The idea that closeness on the interactome relates to phenotypical similarity has applications in disease gene prediction and differential diagnosis [1–4]. For instance, recent methods have successfully exploited these concepts to prioritise candidate disease genes according to their level of connectivity to known disease genes [2, 3, 5–8]. Moreover, the comprehensive study of the phenotypical similarities of diseases can help in understanding their aetiology and reveal commonalities in their pathophysiology.

A few measures have been developed to systematically quantify the similarity between pairs of diseases (see Supplementary Note 1). LanDis relies on the Caniza measure, which summarises the information about diseases that is scattered across the biomedical literature [4]. The method is based on the idea that a disease can be described accurately by the set of MeSH terms used to annotate the publications relevant for that disease. Pairwise similarities between diseases are then calculated by exploiting the structure of the MeSH ontology. A comparison of the different similarity measures using sets of diseases with known disease genes, showed that the Caniza similarity outperforms all other measures in terms of accuracy at predicting closeness of disease modules on the interactome [4]. This is probably due to the large volume of information, i.e., the thousands of disease-related publications, which contribute to the measure. Also notice that the Caniza similarity is related to the human disease network [9] that contains a link representing a similarity between each pair of diseases that share disease genes (the relationship between the Caniza similarity and the human disease network is discussed in Caniza et al. [4], see Supplementary Note 3).

While the importance of disease similarity measures for medical research is clearly understood, until now their use in practice has been limited. An important reason is that disease similarities are mainly available only as matrices containing millions of numerical values, one for each disease pairs, and this limits the scientists’ ability to use this information for reasoning and making inferences.

In this paper, we present LanDis, a freely available web server that provides an intuitive interface to analyse millions of similarity relationships between heritable diseases, together with the evidence supporting such relationships.

## Results

**Fig. 1:**
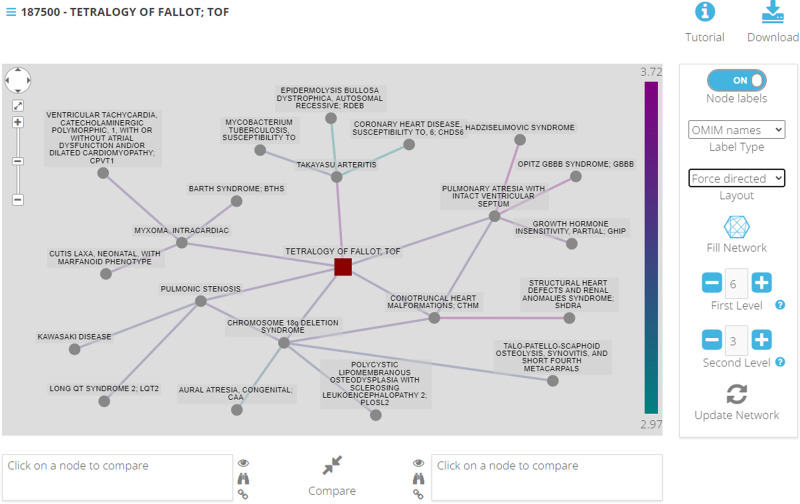
The disease similarity landscape of the congenital heart defect Tetralogy of Fallot, TOF. Each node represents a disease and links are coloured based on the Caniza similarity between the linked diseases. The diseases in the TOF landscape are either directly associated with heart conditions, such as the case of ALGS1 [19], CTHD6 [12] and RAI or indirectly through some common phenotypic features such as DGS.

In LanDis, the similarity landscape is represented as a graph in which nodes are diseases and links are labelled with the Caniza similarity score between the diseases they connect. Figure 1 shows the landscape of the OMIM disease *Tetralogy of Fallot*, TOF (MIM: 187500), represented by the central node in the figure. TOF is a congenital heart defect characterised by a ventricular septal defect, pulmonary valve stenosis, thickened right ventricle and overriding aorta [10]. Patients with TOF develop cyanosis in proportion to the pulmonary valve stenosis, rapid breathing to compensate for low oxygen levels and a heart murmur. Let us analyse each disease that we find connected to TOF in our similarity landscape. The *Conotruncal Heart Malformations* CHTM (MIM: 217095) disorder includes the TOF malformations and is known to be causally related to gene NKX2-5, a gene also known to be causally related to TOF. Both *Alagille Syndrome 1* ALGS1 (MIM:118450) and *Right Atrial Isomerism* RAI (MIM:208530) not only share phenotypic similarities with TOF such as pulmonary stenosis (ALGS1) and complete atrioventricular septal defects (RAI), but also have disease genes in common with TOF, namely JAG1 and GDF1 (ref. [11]). *Congenital heart defects, Multiple Types* CHTD6 (MIM: 613854) (formerly *Transposition of the great arteries* DTGA3) often have ventricular septal defects and associations between CHTD6 and the TOF-associated gene GDF1 have been reported in the literature [12]. *Aortic Arch Interruption, Facial Palsy, Retinal Coloboma* (MIM: 107550) exhibits symptomatic similarities with TOF, such as fatigue, rapid breathing, fast heart rate, low oxygen levels among others [13]. Beyond the symptomatic similarities, TOF shares common physiological features with *Aortic Arch Interruption* (MIM: 107550), such as ventricular septal defects. Finally, *Takayasu Arteritis* (MIM: 207600) is an inflammatory disease of the arteries, with predilection for the aorta and its branches. The disease is characterised by lesions that can, among others, have stenotic qualities [14].

Interestingly, the diseases in the graph without a direct connection to TOF reflect not only their associations with their immediate neighbours but also, to some extent, with TOF. For example, *DiGeorge syndrome* DGS (MIM: 188400) not only shares a gene with TOF (TBX1), but also the outflow tract defects present in DGS are associated with a higher incidence of conotruncal abnormalities [15].

**Fig. 2:**
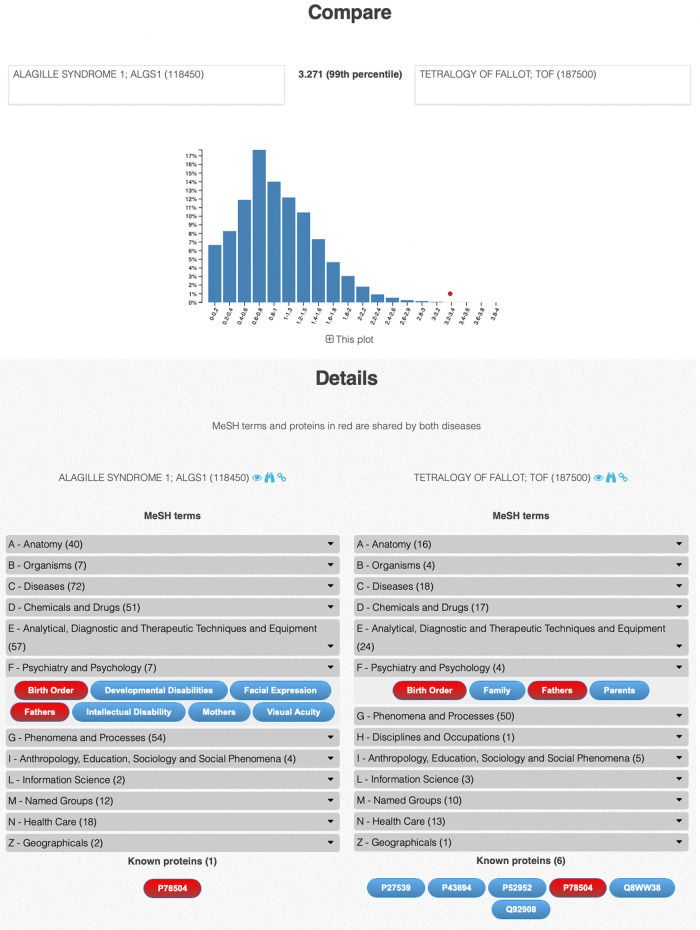
Detailed comparison of TOF and ALGS1. In the histogram, each bar represents the percentage of disease pairs with similarity score in the corresponding range. The red circle indicates the range of the similarity of the TOF–AGS1 pair. The bottom of the figure shows the MeSH annotations grouped by ontology—these are accessible by expanding the collapsed sections. MeSH terms that are common to both diseases are indicated in red. Links to the relevant MeSH term pages and OMIM pages are also available. The known disease genes are linked to their UniProt page.

LanDis is a web application in which the user can interact with all the elements in the graph and the diseases can be repositioned either by dragging them or through several predefined layouts (circular, concentric, grid, breadth-first and force directed). Seamless exploration of the disease’s similarity landscapes can be performed through the selection of any disease in the landscape. Every disease similarity landscape can be downloaded in publication-quality, high-resolution PNG images for offline analysis. Users can also select a disease and obtain a catalogue of those diseases most similar to it in a tabular format, as well as a detailed comparison between pairs of diseases---Fig. 2 shows the *Compare* page for TOF and ALGS1. For users who wish to use the Caniza similarity data as part of a larger pipeline, a CSV plain-text file is available from the download section of the website. To ease further exploration, LanDis links every MeSH term, disease and disease gene to its corresponding entry in the OMIM, UniProt and National Library of Medicine websites, respectively.

## Discussion

LanDis offers a new perspective to explore disease similarity relationships. It is a simple and powerful tool which can be used for differential diagnosis as diseases that present similar molecular features will be assigned high similarity. Importantly, LanDis provides the user with a rationale for the results by making available the set of MeSH terms, corresponding to disease phenotypes, used to calculate the disease similarity. In this way, scientists can focus on the clinical features deemed more critical while concentrating on a selected list of highly similar diseases.

Notably, LanDis is able to find similarities at the molecular level between diseases even in the absence of any molecular information—this is because it only needs a list of publications associated with each disease. Supplementary Fig. 1 shows the number of publications, MeSH terms and genes associated with the diseases in LanDis. As is expected, a disease with many referenced publications tends to be annotated by many MeSH terms, but a high number of publications does not necessarily correspond to a high number of known genes—for example, Huntington’s disease, that has more than 450 references and close to a 1000 MeSH terms, is associated to a single gene. However, since LanDis relies exclusively on publications and their corresponding MeSH terms, the sparseness of molecular information does not prevent the similarity scores from being calculated. In fact, LanDis attempts to encapsulate all available information about diseases—for example, the references of type 2 Diabetes (NIDDM) include information about several clinical trials and multi-year studies on the effects of glucose on insulin levels.

LanDis aims at becoming a support tool for bioinformaticians as well as medical practitioners. It is freely available through its website, no registration or installation is needed and our servers store no information about the users.

## Online methods

### Disease similarities and datasets

LanDis mines OMIM to extract 139,549 PubMed references. For each publication, LanDis queries the Medline API obtaining a total of 17,110 MeSH terms. A few disease entries in OMIM with no references or MeSH annotations are excluded from LanDis, for a working total of 9735 diseases. This amounts to over 44.7 million similarities, one per disease pair.

To produce the pairwise similarities, LanDis relies on the structure of the MeSH ontologies. The similarity between a pair of diseases is given by the Resnik similarity of the sets of MeSH terms annotating the diseases [16]. The Resnik similarity score of two sets of MeSH terms is given by the information content of their lowest common ancestor, which is defined as the negative logarithm of the probability of finding it among the annotations of the OMIM diseases [16–18].

MeSH terms are organised into 16 ontologies and a given disease can be annotated with terms from more than one ontology. This means that for every disease up to 16 similarities can be calculated. Following Caniza et al. [4], LanDis exploits the fact that these ontologies are interconnected to combine them and produce a single score.

### Implementation details

LanDis is implemented using Python and the Django framework, following a strict Model-View-Controller architecture. The data persistence is provided by a single-file SQLite database, which holds the similarity data and all additional information required to provide LanDis functionalities. Indices where defined to improve access time to the SQL database. The user interface was designed using HTML 5 and the JQuery JavaScript library. Additionally, two well-known JavaScript libraries, D3.js and Cytoscape.js, are included. D3.js provides the tools for dynamic visualisations of the similarity data and Cytoscape.js provides the engine for LanDis disease landscape explorer. This allows for a flexible interface that fits most resolutions for desktops, laptops and most mobile devices.

There are no special requirements for a user’s computer, since all user-side JavaScript code was carefully developed to reduce its footprint. Warnings are displayed for larger more resource-consuming plots, allowing the user to choose whether to continue with the operation.

The source code is freely available from GitHub at https://github.com/paccanarolab/landis and is released under the GPLv3 license. We have tested LanDis on all major browsers and operating systems (mobile and desktop), and it performs best on Google Chrome. A comprehensive user manual is included in Supplementary Note 2.

### Supplementary information

Supplementary Material

## Data Availability

The disease similarity between all diseases calculated for this study is available to download from our website: https://paccanarolab.org/static_content/disease_similarity/combined_similarity_triplet_2023.zip. The OMIM to MeSH mapping is also available: https://paccanarolab.org/static_content/disease_similarity/mim2mesh_2023.tsv. The data that support the findings of this study are available from OMIM but restrictions apply to the re-distribution of these data, which were used under license for the current study, and so are not publicly available.
