# Supplementary material for: LanDis: the disease landscape explorer
Source: Eur J Hum Genet. 2024 Jan 10;32(4):461–5. doi: 10.1038/s41431-023-01511-9 (PMC10999415; doi:10.1038/s41431-023-01511-9)
Supplement: Supplementary file 1 — Supplementary Material [file 41431_2023_1511_MOESM1_ESM.docx]

Supplementary Information for LanDis: The Disease Landscape Explorer

**Horacio Caniza, Juan J. Cáceres, Mateo Torres, Alberto Paccanaro**

Table of Contents

[Note 1: Quantifying similarities between diseases 2](#_Toc149735349)

[Supplementary Figure 1: Information used by LANDIS. 4](#_Toc149735350)

[Note 2: LanDis user manual 5](#_Toc149735351)

[Explore 5](#_Toc149735352)

[Search 9](#_Toc149735353)

[Compare 11](#_Toc149735354)

[UX/UI testing 13](#_Toc149735355)

[Note 3: Human Disease Network and Disease similarity network 15](#_Toc149735356)

[References 18](#_Toc149735357)

# Note 1: Quantifying similarities between diseases

A few measures have been developed to systematically quantify the similarity between pairs of diseases (1-6). van Driel *et al.* (1) proposed a text-mining analysis of OMIM (9), whereby diseases are classified based on features retrieved from the *Clinical Synopsis* field in OMIM. The authors use a subset of MeSH to build a feature vector for each disease. These vectors are then compared to obtain a measure of the similarity between diseases. Robinson *et al.* (2) proposed and built the Human Phenotype Ontology, a manually curated ontology that contains terms for the description of phenotypic abnormalities for diseases in OMIM. The similarity between diseases is then computed using an information based measure between sets of terms representing diseases on the ontology. Zhou et al. (3) mine PubMed, extracting the MeSH terms associated to each publication and analyse the co-occurrence of a symptom term (terms in the C23 ontology in MeSH) and a disease term (terms in the C01-C26, except for the C22 and C23 ontologies in MeSH). This co-occurrence is compiled into a feature vector that characterises each disease based the frequency of its symptoms across PubMed. Similarity between every disease is obtained by computing the cosine of the angle between the feature vectors and then conserving only those statistically significant scores. The work of Park *et al.* (4) builds on the premise that diseases whose proteins share a common subcellular localisation are phenotypically related. Similarity between two diseases is determined by an association score between diseases based on the cellular co-localisation of their disease proteins. Cheng et al. (5) calculate disease similarity using disease-related gene sets in a weighted network of human gene function. Next semantic similarity is devised to calculate disease similarity using the relationship between two diseases from Disease Ontology. Both types of similarities are then integrated to measure disease similarity. Hoehndorf et al. (6) use several ontologies to retrieve the co-occurrence of various phenotypes, which they combine with known disease genes from OMIM, the significance of such co-occurrences is then ranked using several metrics to build a similarity score based on the simSIC measure. Finally, Caniza et al. (7) compare the MeSH terms used to annotate the publications relevant to a pair of diseases. Semantic similarity measures on the MeSH ontology itself are used to determine the similarity between a pair of diseases.

***
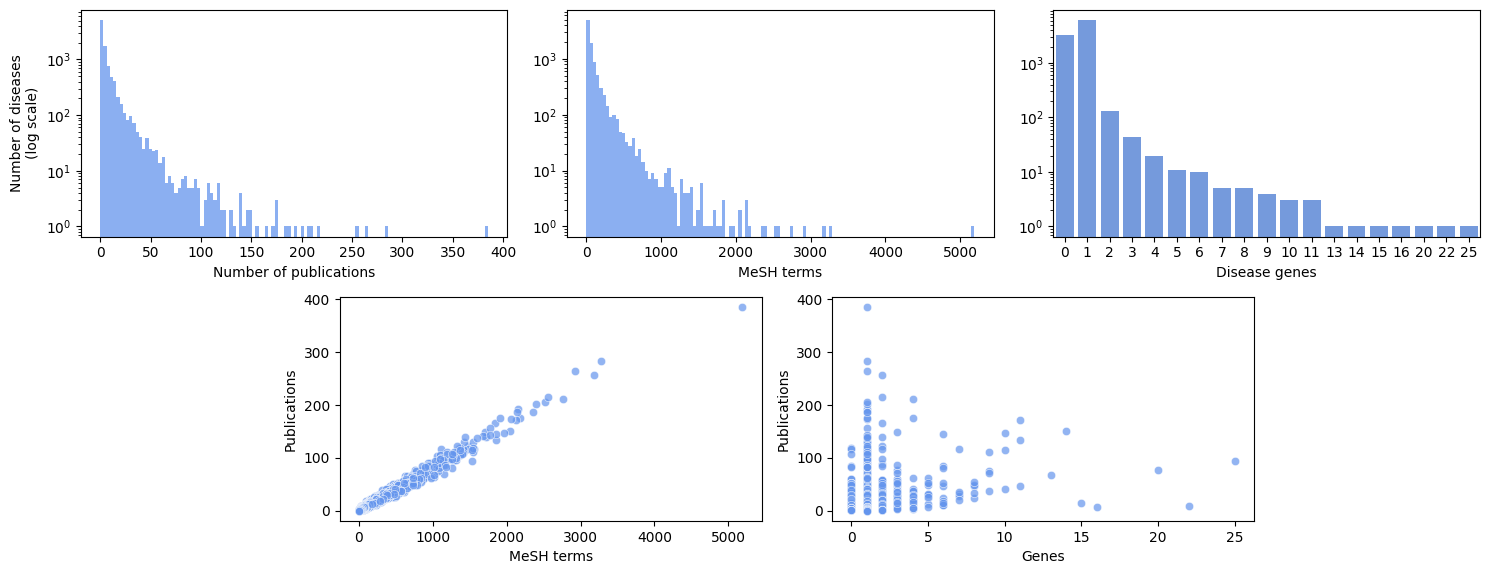
***

# Supplementary Figure 1: Information used by LANDIS.

***(Top left)*** *Number of publications associated to OMIM diseases. On average, diseases have 8.1 references (standard deviation 16.1).* ***(Top middle)*** *Number of MeSH terms associated to OMIM diseases. The average number of MeSH terms is 100.3 (standard deviation 197.4).* ***(Top right)*** *Number of disease genes per OMIM disease. On average, diseases have 0.7 genes (standard deviation 0.84).* ***(Bottom Left)*** *Diseases with many references tend to be annotated with many MeSH terms (0.99 correlation).* ***(Bottom Right)*** *Many publications do not necessarily imply many known disease genes (0.29 correlation). In the scatter plots each dot corresponds to a disease.*

# Note 2: LanDis user manual

The LanDis web tool is composed of 3 main pages:

- Explore: This page arranges diseases in a network view, where each disease is a node, and the links are the similarities between them. It is centred on one disease, and by default the local neighbourhood of that disease is displayed.
- Search: For a given disease, this page shows a table with including 100 most similar diseases, sorted by decreasing similarity scores.
- Compare: This page compares two diseases to one another, showing their similarity score in context to the overall distribution of disease-disease similarities contained in the database.

Here, we explain the interface of each component.

## Explore

The most prominent in this page is the Network view (shown in Supplementary Figure 2.1 below). The interactive network visualisation panel (A) is centred on the selected disease, which is represented as a crimson square node. Other diseases in the neighbourhood are represented as circles. The edges between these nodes represent the calculated similarity score. A colour bar on the side provides a visual reference of the range of similarity scores present in the visualisation panel. Directly below this panel, two fields (B and C) are present to allow users to conveniently compare two diseases or navigate to other related pages centered on that disease. If fields B and C are not populated, the user has two ways to populate them for a comparison:

- Click on a node. This will change its colour to yellow, and the name and OMIM identifier will appear on field B. Then, click on a different node, which will also change its colour to yellow, and will populate field C.
- Click on an edge between any pair of nodes. This will populate both B and C with the information of the two diseases connected by the edge.

Once the fields are populated, the compare button will redirect the user to the Compare page between the populated diseases. Additionally, the user may navigate to the Explore page centred on the selected disease by clicking on the eye icon next to the field. Clicking on the binocular icons will take the user to the Search page for the selected disease. Finally, clicking on the chain icon will take the user to the OMIM page for the selected disease.


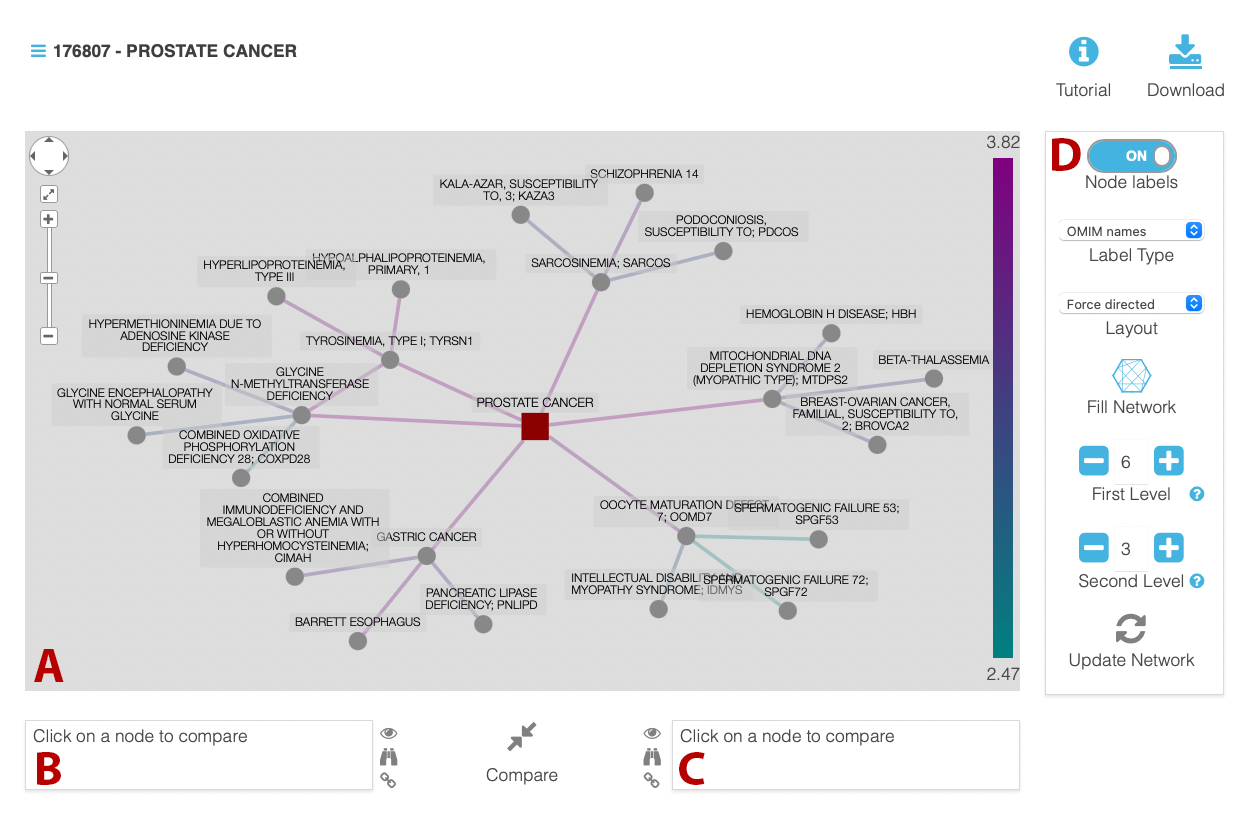


***Supplementary Figure 2.1*** *Network view of the Explore page. A) The interactive network shows the selected disease as a red square in the centre of the network. Diseases in the neighbourhood are shown as circle nodes. Edges between the nodes in the network represent the similarity between diseases and are coloured according to the similarity score. On the right side of this panel, a colour bar includes a visual reference to map the colour of the edge to the actual score. B) Once a node is clicked in the interactive panel, the field in B will be populated with the disease the clicked node represents. C) Once a second node is clicked. The field on the right will be populated with the disease represented by the second node clicked. D) The control panel on the right-hand side contains multiple controls to adapt the visualisation in the interactive panel. Clicking on an edge will populate fields B and C simultaneously. Regardless of the selections in fields B and C, clicking on an empty area of the network pane will remove all selections. If two nodes are already selected and a third node is clicked, the previously selected diseases will be removed, and the clicked disease will populate field B.*

The second element in this page, below the Network view is the table with details (shown in Supplementary Figure 2.2 below). The name and OMIM identifier are shown at the top, and clicking on the chain icon will take the user to the OMIM website for the selected disease. Then, a table with all the MeSH terms associated to this disease are included. The table is organized using the MeSH hierarchical categories. The user can expand each section individually or click on the “Expand all” button. Every MeSH term associated with the disease is represented as a blue button that, when clicked, redirects the browser to the MeSH website for that term. Finally, if any protein is known to be associated to the selected disease, they will be included below the MeSH table. Proteins will be shown as blue buttons as well that, when clicked, will redirect to the UniProt entry for that specific protein.


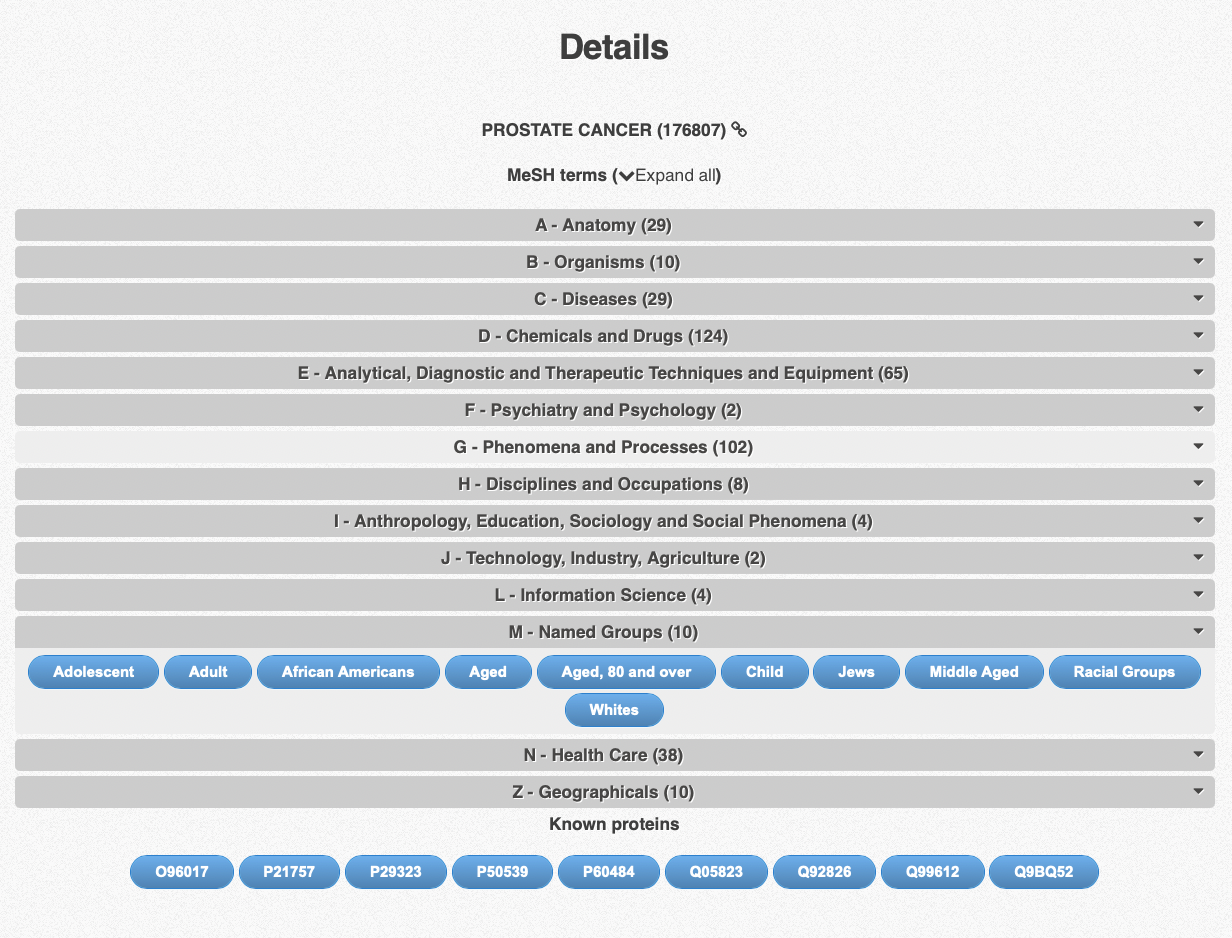


***Supplementary Figure 2.2*** *The Details table. This interactive table is located below the interactive network visualization in the Explore page. It includes a table of all MeSH terms associated with a disease, organized in collapsible sections. At the bottom, if any protein is known to be associated with the disease, it will be listed. All listed elements are depicted as blue buttons that, when clicked, redirect to the MeSH or UniProt websites for MeSH terms and proteins respectively.*

## Search

The Search page includes the top 100 most similar diseases when compared to the selected disease (shown in Supplementary Figure 2.3 below). The name and OMIM identifier are prominently displayed at the top of the page. Users may click on the eye icon to go to the Explore page described in the previous section centred in the selected disease. Clicking on the square icon will instead take the user to the OMIM website for that disease.

Then, a table follows with 100 diseases ordered by decreasing similarity score to the selected disease (only 3 displayed in Supplementary Figure 2.3). For each listed disease we show:

- Its place in the list (Order)
- OMIM identifier. Clicking on the number itself will take the user to the Search page for that disease, clicking on the icon with two arrows will take the user to the compare page between the disease shown in the title and the disease of that row in the table. Clicking on the eye icon takes the user to the Explore page centred on the disease of that row. Clicking on the chain icon takes the user to the OMIM website for the corresponding disease.
- The name of the disease
- The similarity score between the disease in the row and the disease shown at the top.
- Proteins associated with the disease at the relevant row. Clicking the square icon takes the user to the UniProt page for that protein.

The user has the option to download this table to their computed by clicking on the download icon on the top right of the page.


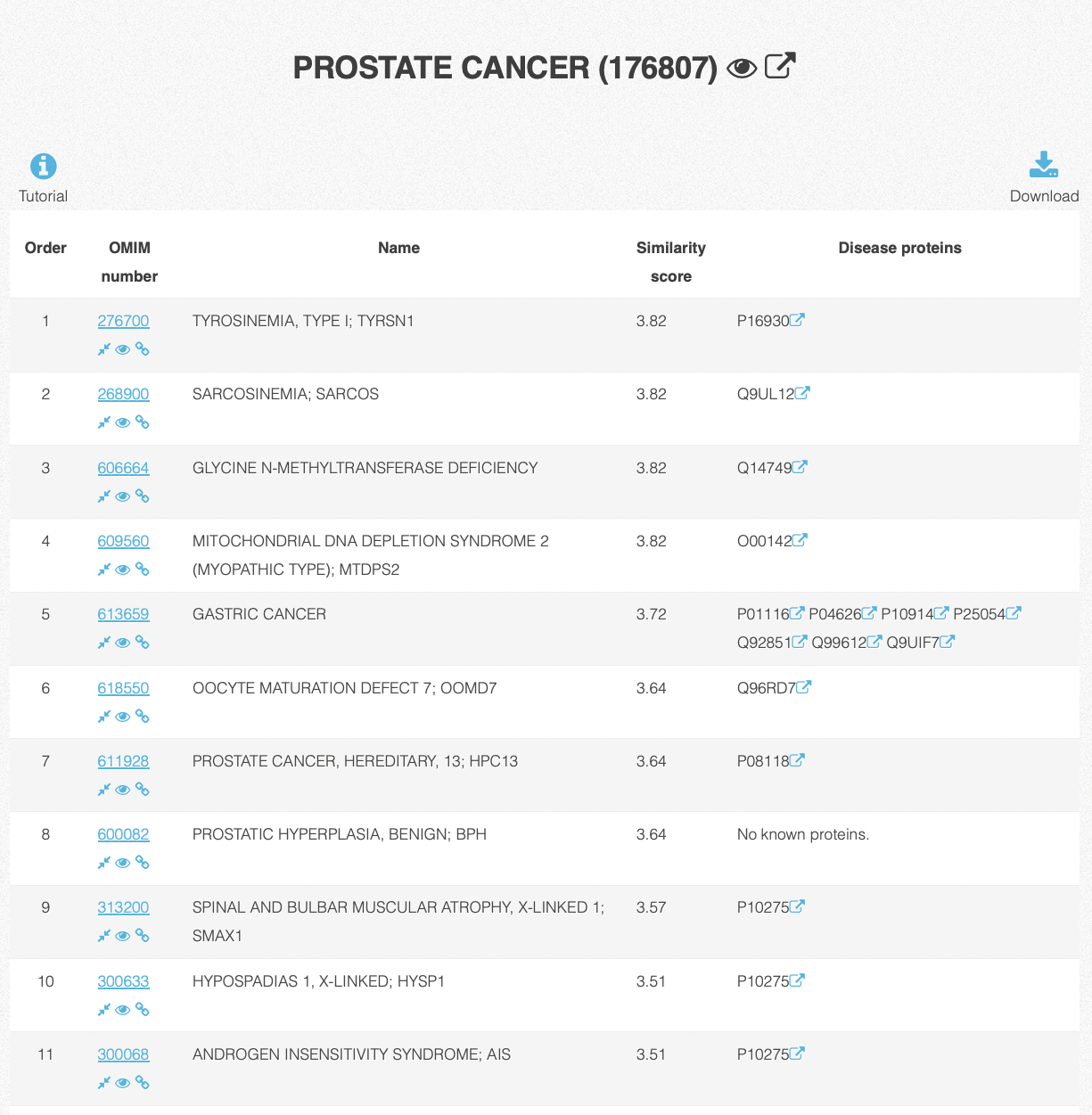


***Supplementary Figure 2.3*** *the Search table shows the 100 most similar diseases to the selected disease, alongside other relevant information. Links in the table take the user to relevant pages such as OMIM and UniProt, and the user may download the table as well.*

## Compare

The Compare page begins by putting the similarity score between the two diseases being compared to the overall distribution of scores contained in the entire LanDis database (shown in Supplementary Figure 2.4 below). At the top of the page, the two diseases are displayed together with their OMIM identifiers, and the similarity score is display between them. A histogram with the distribution of similarity scores is then presented, and a red dot is placed on the bin that includes the score between the two selected diseases.

**
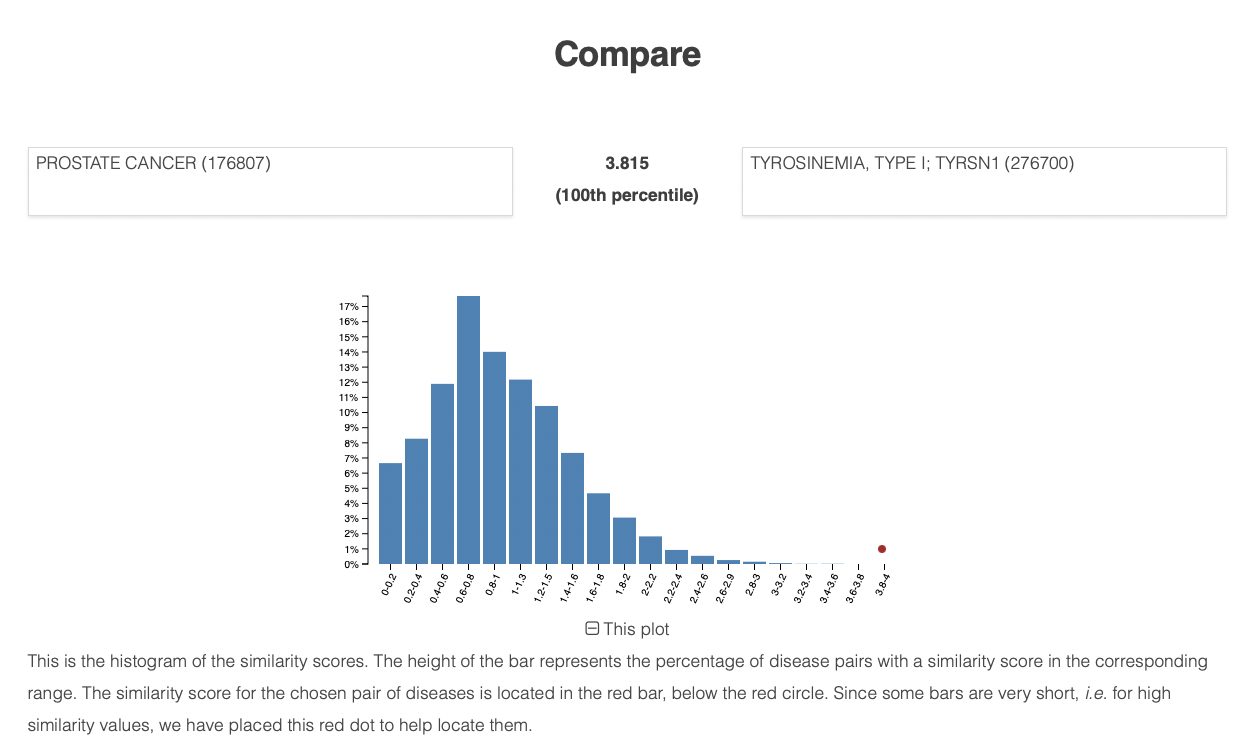
**

***Supplementary Figure 2.4*** *The Compare view starts by putting the similarity score between two diseases in context. A histogram displays where the score between the two selected diseases falls into the overall distribution of scores.*

The second part is a table similar to the one in the Search Page, but this one additionally highlights the MeSH terms and disease proteins shared by the two diseases, displaying them as red buttons (see Supplementary Figure 2.5 below).

**
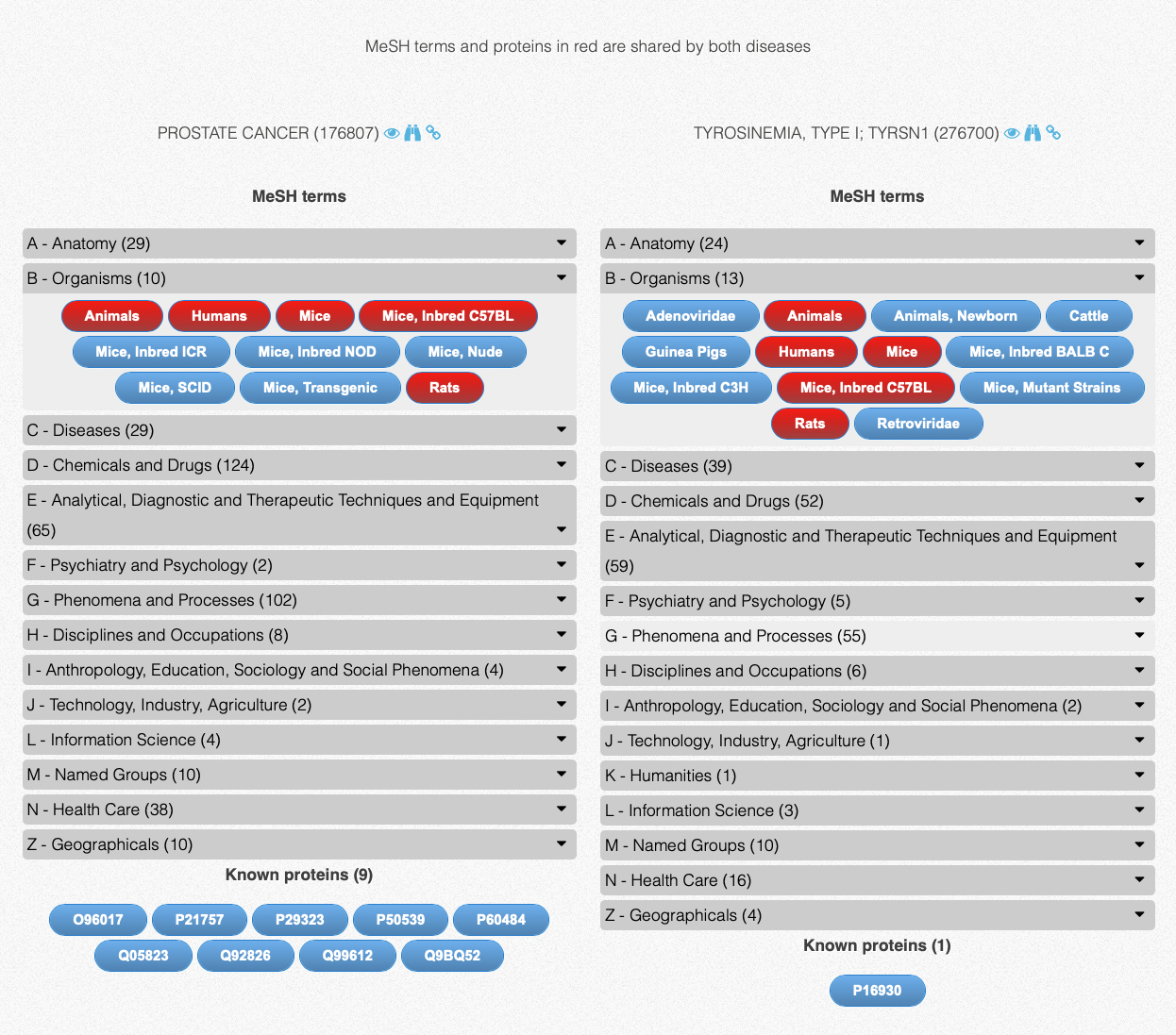
**

***Supplementary Figure 2.5*** *The compare page includes a table that allows a side by side comparison of the MeSH terms and proteins associated disease proteins. Shared elements are displayed in red.*

## UX/UI testing

All pages in LanDis where tested across multiple browsers and operating systems, as detailed below:

- Browsers tested:
  - Mozilla Firefox 118.0.2
  - Opera 103.0.4928.16
  - Google Chrome 118.0.5993.70/71
  - Safari 17.0
  - Microsoft Edge 17.0.2045.60
- Operating Systems used for the testing:
  - Windows 11
  - Windows 10
  - macOS 14.0
  - macOS 13.6
  - Linux (*note:* ***Safari*** *was not tested in these operating systems*):
    - Ubuntu 23.04
    - Ubuntu 22.04
    - Linux Mint 21.2
    - Fedora 38
    - Arch Linux 2023.09.01

# Note 3: Human Disease Network and Disease similarity network

The Barabási diseasome is conceptualized as a bipartite graph, consisting of two sets of nodes: diseases and genes. A connection exists between a disease node and a gene node if the gene is associated with the respective condition, according to the Online Mendelian Inheritance in Man (OMIM) database (9). The authors used the diseasome graph to create two distinct network projections. The first is the Human Disease Network, a weighted network wherein links denote the number of shared genes between pairs of diseases. The second is a disease gene network, a binary network that connects two genes if they are both associated with the same disease.

While the disease similarity network shown in LanDis shares similarities with the Human Disease, links in LanDis are defined by the phenotypic similarity between diseases. This encompasses the physical manifestations of the disease, which include signs, symptoms, drugs known to have an effect on the disease, among other factors. This expansive characterization of disease forms the basis of our measure, enabling the comparison between any two diseases, regardless of whether their genetic bases are known.

The disease similarity network stems from a network-based perspective of human disease. Accordingly, a disease, with its intricate and variable symptoms, arises from disturbances in the underlying molecular machinery of the human body. By grounding our associations in phenotypic descriptions, we create a framework that allows for a more comprehensive linkage of diseases, and without assumptions about the nature of the phenotype-genotype relationship.

The Caniza disease similarity can be used to produce a 3D graphical representation of human diseases automatically. Supplementary figure 3 shows the embedding of diseases into 3D space obtained applying t-SNE, a well-known dimensionality reduction technique.


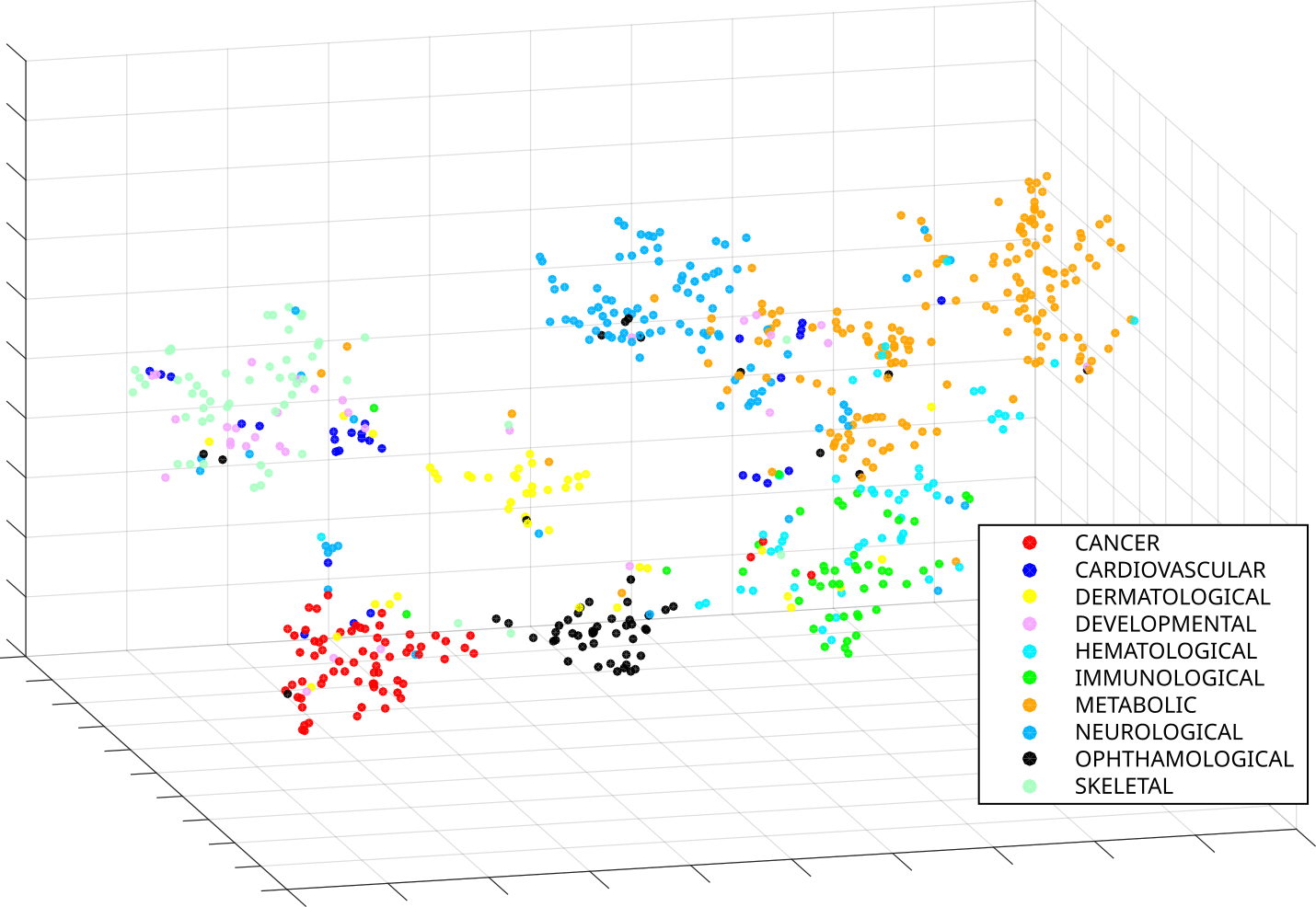


***Supplementary Figure 3.*** *Embedding of hereditary diseases in 3D space based on the Caniza disease similarity using t-SNE. Each point represents an OMIM disease. Colours are assigned based on their disorder class according to Goh et al.(8). The figure shows the diseases belonging to the 10 most numerous disease classes in Goh et al. (8).*

# References

1. Van Driel MA, Bruggeman J, Vriend G, Brunner HG, Leunissen JA. A text-mining analysis of the human phenome. European journal of human genetics: EJHG. 2006;14(5):535.

2. Robinson PN, Mundlos S. The Human Phenotype Ontology. Clinical Genetics. 2010;77(6):525-34.

3. Zhou X, Menche J, Barabási A-L, Sharma A. Human symptoms–disease network. Nature communications. 2014;5:4212.

4. Park S, Yang JS, Shin YE, Park J, Jang SK, Kim S. Protein localization as a principal feature of the etiology and comorbidity of genetic diseases. Molecular systems biology. 2011;7(1):494.

5. Cheng L, Li J, Ju P, Peng J, Wang Y. SemFunSim: a new method for measuring disease similarity by integrating semantic and gene functional association. PLoS ONE. 2014;9, e99415.

6. Hoehndorf R, Schofield PN, Gkoutos GV. Analysis of the human diseasome using phenotype similarity between common, genetic, and infectious diseases. Scientific Reports. 2015;5:10888.

7. Caniza H, Romero AE, Paccanaro A. A network medicine approach to quantify distance between hereditary disease modules on the interactome. Scientific reports. 2015;5.

8. Goh, K. I., Cusick, M. E., Valle, D., Childs, B., Vidal, M., & Barabási, A. L. (2007). The human disease network. Proceedings of the National Academy of Sciences, 104(21), 8685-8690.

9. Online Mendelian Inheritance in Man, OMIM®. McKusick-Nathans Institute of Genetic Medicine, Johns Hopkins University (Baltimore, MD), {date}. World Wide Web URL: https://omim.org/
